# Image-Guided Adaptive Brachytherapy Using Patient-Specific 3D-Printed Templates for Complex Locally Advanced Cervical Cancer: A Real-World Implementation Study

**DOI:** 10.3390/cancers18152399

**Published:** 2026-07-25

**Authors:** Yuanjie Cao, Imashi Sandupama Wickramage, Chen Li, Youheng Tan, Wenwen Zhang, Qingsong Pang, Jie Chen

**Affiliations:** 1 Department of Radiation Oncology, Tianjin Medical University Cancer Institute & Hospital, Tianjin’s Clinical Research Center for Cancer, Key Laboratory of Cancer Prevention and Therapy, National Clinical Research Center for Cancer, Tianjin 300060, China; lichen.radonco@tmu.edu.cn (C.L.); zhangwenwen@tjmuch.com (W.Z.); pangqingsong@tjmuch.com (Q.P.); 2 Department of Radiation Oncology, Tianjin Medical University Cancer Institute & Hospital, Tianjin Medical University, Tianjin 300060, China; imashisandupama@gmail.com; 3 School of Automation, Central South University, Changsha 410083, China; tyheng@163.com

**Keywords:** cervical cancer, image-guided adaptive brachytherapy, adaptive radiotherapy, 3D-printed template, intracavitary/interstitial brachytherapy, curved-channel guidance, real-world implementation, dosimetry

## Abstract

**Background/Objectives:** Image-guided adaptive brachytherapy is a core component of definitive treatment for locally advanced cervical cancer (LACC). However, implantation remains challenging in bulky, asymmetric, or anatomically complex tumors, where standard applicator geometry or purely straight interstitial trajectories may be insufficient for individualized target coverage. This study evaluated the real-world implementation of a patient-specific 3D-printed template-guided adaptive brachytherapy workflow for complex LACC. **Methods:** We retrospectively reviewed 120 consecutive patients with FIGO 2018 stage IB3–IVA cervical cancer treated with definitive chemoradiotherapy followed by high-dose-rate image-guided brachytherapy between March 2023 and March 2025. All patients were treated using a patient-specific 3D-printed template-guided hybrid intracavitary/interstitial workflow integrating CT/MRI-based target assessment, individualized catheter trajectory planning, template fabrication, implantation verification, and adaptive treatment planning. Straight-channel or curved-channel guidance was selected according to residual tumor geometry and pelvic anatomy, with flexible plastic interstitial catheters used for curved or anatomically constrained trajectories. Procedural deliverability, dosimetry, toxicity, early clinical outcomes, and exploratory dose–outcome patterns were analyzed. **Results:** The median HR-CTV volume was 55.9 cm^3^, and the median HR-CTV D90 was 92.9 Gy EQD2. Median organ-at-risk D2cc values remained within contemporary institutional and guideline-consistent constraints. A total of 555 template-guided HDR brachytherapy fractions were delivered. The median applicator-and-catheter placement time was 4.21 min per fraction, with a median of 7.25 implanted channels. Minor and major insertion-related bleeding occurred in 10.8% and 1.7% of patients, respectively. At a median follow-up of 20.1 months, estimated 3-year overall survival, progression-free survival, local recurrence-free survival, regional recurrence-free survival, and distant metastasis-free survival were 77.9%, 76.8%, 94.3%, 98.0%, and 86.2%, respectively. Late grade ≥ 3 gastrointestinal and genitourinary toxicities occurred in 2.5% and 1.7% of patients, respectively, with no grade 4–5 events. Exploratory dose–outcome analyses suggested hypothesis-generating dose–outcome patterns, but these findings were not intended to define or validate a clinical dose threshold. **Conclusions:** This real-world implementation study supports the feasibility of patient-specific 3D-printed template-guided adaptive brachytherapy for complex LACC. By translating CT/MRI-based individualized trajectory planning into template-guided intracavitary/interstitial catheter placement, this workflow achieved guideline-consistent target coverage, acceptable organ-at-risk doses, efficient procedural delivery, and low severe toxicity within the available follow-up. Dose–outcome findings remain exploratory and require validation in more mature cohorts.

## Simple Summary

Image-guided adaptive brachytherapy is an essential part of curative treatment for locally advanced cervical cancer, but accurate implantation can be difficult in patients with bulky, asymmetric, or anatomically complex tumors. In these cases, standard applicators or purely straight needle paths may not adequately cover the residual tumor while protecting nearby organs such as the bladder, rectum, sigmoid colon, and bowel. In this study, we evaluated a real-world clinical workflow using patient-specific 3D-printed templates to guide individualized intracavitary/interstitial brachytherapy. The template was designed from imaging before brachytherapy and used to translate planned catheter trajectories into a reproducible implantation procedure. Curved-channel guidance with flexible plastic interstitial catheters was used when straight paths were insufficient for difficult target regions. In 120 consecutive patients, this workflow achieved guideline-consistent target coverage, acceptable organ-at-risk doses, efficient applicator and catheter placement, and low rates of severe toxicity during the available follow-up. These findings support the practical clinical implementation of patient-specific template-guided adaptive brachytherapy for complex cervical cancer.

## 1. Introduction

Cervical cancer remains a major global health problem, with most patients in developing regions presenting with locally advanced disease [1]. Definitive chemoradiotherapy followed by high-dose-rate image-guided adaptive brachytherapy is a cornerstone of curative treatment for locally advanced cervical cancer (LACC) [2]. By integrating three-dimensional target definition, dose–volume histogram-based planning, and repeated adaptation to tumor regression and pelvic anatomy, image-guided adaptive brachytherapy has substantially improved the therapeutic ratio compared with historical two-dimensional approaches.

The GEC-ESTRO recommendations and ICRU Report 89 established standardized MRI- and CT-based concepts for target delineation, including residual gross tumor volume, high-risk clinical target volume (HR-CTV), and organs at risk (OARs), as well as dose–volume reporting parameters such as HR-CTV D90 and OAR D2cc [3,4,5,6]. These concepts provided the foundation for contemporary image-guided adaptive brachytherapy and enabled multicenter benchmarks and paradigm-shifting clinical evidence, including RetroEMBRACE, EMBRACE-I, and EMBRACE-II, which linked individualized target coverage with improved pelvic control, standardized planning aims, and acceptable late morbidity [7,8,9,10,11]. In this framework, adaptive brachytherapy is not only a planning technique but also a clinical workflow that must convert evolving imaging information into safe, reproducible, and anatomically appropriate applicator and catheter placement.

Despite these advances, implantation remains one of the most challenging steps in complex LACC. Intracavitary applicators alone may be insufficient for bulky, asymmetric, parametrially extensive, or anatomically distorted tumors, especially when residual disease extends laterally, obliquely, or close to dose-limiting OARs [12,13,14,15,16,17]. Conventional interstitial approaches can improve target coverage, but purely straight catheter trajectories may not always provide optimal access to eccentric target extensions without increasing the risk of OAR traversal, excessive tissue trauma, or suboptimal geometry. Therefore, an important implementation gap in modern adaptive brachytherapy is how to translate individualized image-based trajectory planning into a practical implantation procedure that remains efficient and reproducible in routine clinical practice.

Patient-specific three-dimensional printing provides a potential solution to this gap. Rather than functioning as a generic perineal template, a patient-specific 3D-printed template can serve as a physical translation interface between CT/MRI-based pre-planning and intraoperative intracavitary/interstitial catheter placement. By defining individualized channel entry points, directions, depths, and spatial relationships with the intracavitary applicator, such templates may improve geometric reproducibility and reduce the dependence on free-hand intraoperative judgment. In selected anatomically complex cases, curved-channel or oblique guidance using flexible plastic interstitial catheters may further expand the spatial reach of hybrid intracavitary/interstitial brachytherapy toward target regions that are difficult to access with purely straight metallic needle paths.

Early technical and institutional experiences have described the feasibility of 3D-printed templates and curved-channel catheter systems for bulky or parametrially extensive cervical tumors [18,19,20,21,22,23,24,25,26,27,28]. However, evidence remains limited regarding how such patient-specific template-guided adaptive workflows perform when implemented routinely in consecutive real-world patients. In particular, additional data are needed on procedural deliverability, catheter-guidance strategy, target and OAR dosimetry, early clinical outcomes, toxicity, and quality assurance in a high-volume clinical setting.

Therefore, this study aimed to evaluate the real-world implementation of image-guided adaptive brachytherapy using patient-specific 3D-printed templates for complex LACC. We focused on whether an integrated workflow combining CT/MRI-based target assessment, individualized straight-channel or curved-channel catheter guidance, template-guided implantation, post-implant verification, and adaptive treatment planning could achieve guideline-consistent dosimetry, efficient procedural delivery, acceptable early safety, and favorable early pelvic control. Exploratory dose–outcome analyses were additionally performed as hypothesis-generating assessments rather than as definitive threshold validation.

## 2. Materials and Methods

### 2.1. Study Design and Patient Cohort

**Figure 1:**
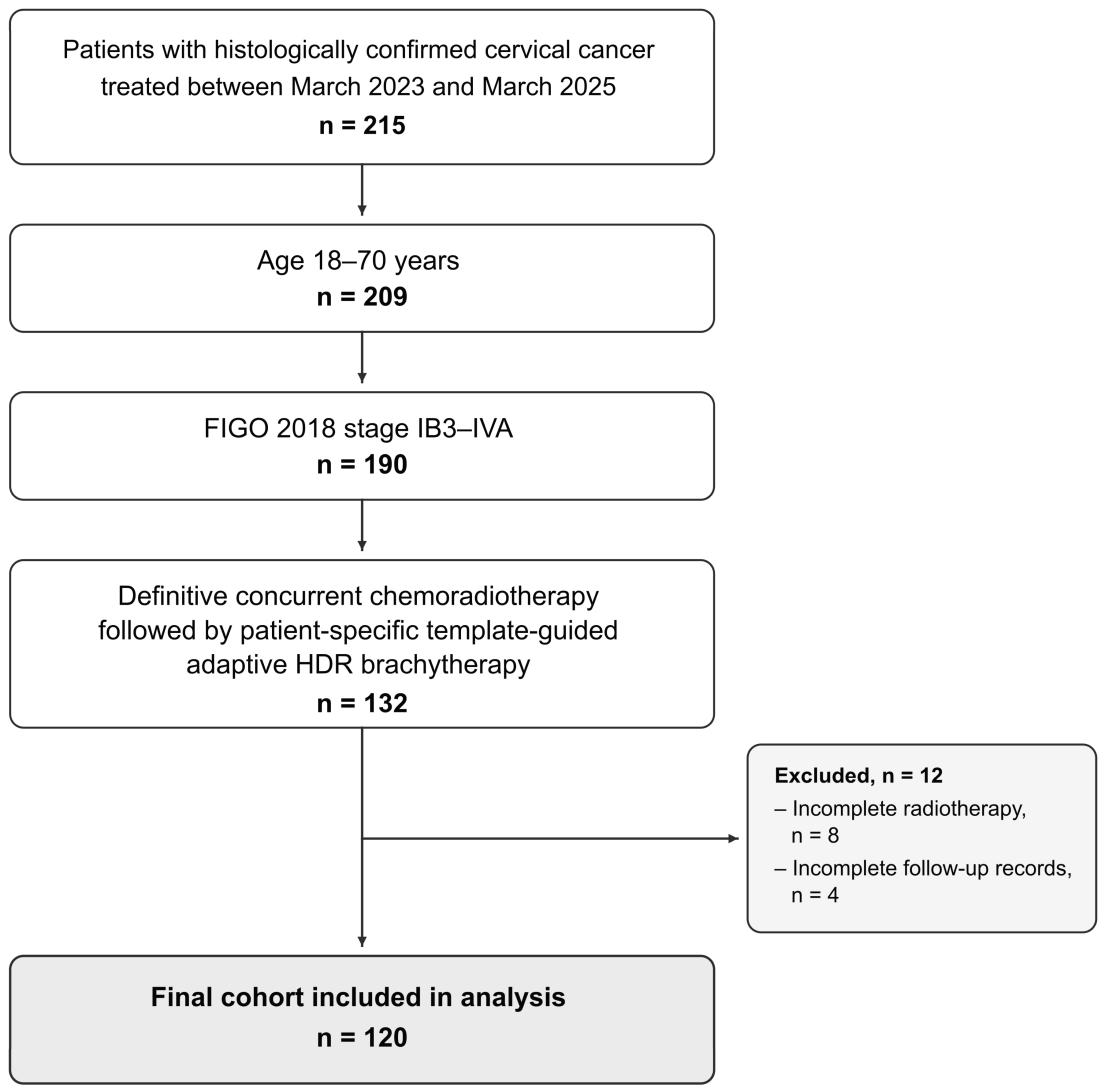
Patient selection flowchart for the study cohort. Flow diagram showing patient screening, eligibility assessment, exclusions, and final cohort inclusion for this retrospective single-center study of image-guided adaptive brachytherapy using patient-specific 3D-printed templates for complex locally advanced cervical cancer.

This retrospective study included 120 consecutive patients with histologically confirmed locally advanced cervical cancer (LACC) treated with definitive chemoradiotherapy followed by high-dose-rate (HDR) image-guided brachytherapy at Tianjin Medical University Cancer Institute & Hospital between March 2023 and March 2025. All patients were staged using the FIGO 2018 system. Exclusion criteria were incomplete radiotherapy, missing imaging required for brachytherapy planning, or loss to follow-up before initial reassessment. The study was approved by the institutional review board, with informed consent waived owing to the retrospective design. The patient selection process is summarized in Figure 1.

### 2.2. External Beam Radiotherapy and Concurrent Chemotherapy

All patients received pelvic external beam radiotherapy (EBRT) using volumetric modulated arc therapy (VMAT) or intensity-modulated radiotherapy (IMRT). A total pelvic dose of 45–50.4 Gy in 25–28 fractions was delivered, corresponding to an EQD2 of approximately 45–49.6 Gy for tumor tissue (α/β = 10). Extended-field irradiation to the para-aortic or common iliac regions was offered for radiologically positive nodal disease according to institutional criteria aligned with contemporary EMBRACE-II principles [29]. Simultaneous integrated nodal boost was delivered when clinically indicated. Concurrent chemotherapy consisted of weekly cisplatin at 40 mg/m^2^ unless contraindicated. Treatment interruptions were minimized whenever possible.

### 2.3. Patient-Specific Template-Guided Adaptive Brachytherapy Workflow

High-dose-rate brachytherapy was delivered using an image-guided adaptive workflow based on patient-specific 3D-printed template-guided hybrid intracavitary/interstitial (IC/IS) implantation. All patients were treated within this template-guided framework. The workflow integrated pre-brachytherapy imaging, CT/MRI-based target assessment, individualized catheter trajectory planning, patient-specific template fabrication, applicator and catheter placement, post-implant imaging verification, and adaptive treatment planning.

Before brachytherapy, pelvic MRI, primarily T2-weighted sequences, was reviewed together with planning CT to assess residual tumor extent, uterine position, parametrial or vaginal extension, and the spatial relationship between the HR-CTV and adjacent organs at risk. For each patient, an intracavitary applicator was selected according to uterine anatomy and residual tumor geometry. Interstitial catheter guidance was then individualized within the patient-specific template. Straight-channel guidance was used when a linear metallic needle trajectory was considered sufficient for target coverage. Curved-channel or oblique guidance was selected when eccentric, lateral, parametrial, vaginal, or anatomically constrained target extensions were unlikely to be reached safely by a purely straight trajectory.

MRI and CT provided complementary information within the adaptive brachytherapy workflow. T2-weighted pelvic MRI was used to evaluate residual cervical tumor, parametrial or vaginal extension, uterine anatomy, and to define the HR-CTV, whereas CT was used after implantation for applicator verification, catheter reconstruction, OAR assessment, and dose calculation. Intravenous contrast was not routinely administered for brachytherapy planning CT or MRI. PET/CT and ultrasound were not used as primary routine planning modalities in this workflow, although each may provide clinically useful information in selected settings. Within the GEC-ESTRO/ICRU framework, non-contrast T2-weighted MRI provided sufficient soft-tissue information for residual tumor assessment and HR-CTV definition.

Patients were usually assessed for brachytherapy qualification during the last week of EBRT. Pre-brachytherapy MRI and clinical examination were reviewed to evaluate residual disease, parametrial or vaginal extension, OAR relationships, and the feasibility of template-guided catheter placement. Template design and fabrication were generally initiated during the final week of EBRT to allow brachytherapy to begin promptly after EBRT completion. The first HDR fraction was typically delivered within 1–5 days after EBRT completion when blood counts and clinical conditions were acceptable. No routine waiting period was imposed unless blood counts, infection, bleeding, or clinically significant necrosis raised safety concerns.

Flexible plastic interstitial catheters, primarily ProGuide^®^ plastic needles (Elekta, Veenendaal, The Netherlands), were used for curved-channel or anatomically constrained catheter placement. These catheters were not treated as a separate competing technique but as one component of the same patient-specific adaptive implantation workflow. The choice of straight-channel or curved-channel guidance was made according to tumor geometry, pelvic anatomy, and anticipated dosimetric requirements rather than by random allocation. Therefore, comparisons between catheter-guidance strategies were considered descriptive and were not intended to establish superiority of one strategy over another.

After applicator and catheter placement, post-implant CT was acquired for applicator verification, catheter reconstruction, OAR assessment, and treatment planning. MRI was used for pre-brachytherapy target assessment and CT/MRI fusion-based adaptive target definition. Post-implant applicator verification and catheter reconstruction were performed on CT. Patients with metallic interstitial needles in situ were not scanned with MRI. When MRI information was incorporated into post-implant planning, it was based on pre-implant or fraction-specific MRI obtained under MRI-compatible conditions and fused to the planning CT to support HR-CTV localization. Plans were optimized for each fraction to account for tumor regression, applicator geometry, catheter position, and anatomical changes.

MRI/CT registration was performed primarily using rigid registration based on the uterus, cervix, applicator relationship, and adjacent pelvic anatomy, followed by physician review and manual adjustment when needed. Deformable registration was not routinely used for dose accumulation in this study.

**Figure 2:**
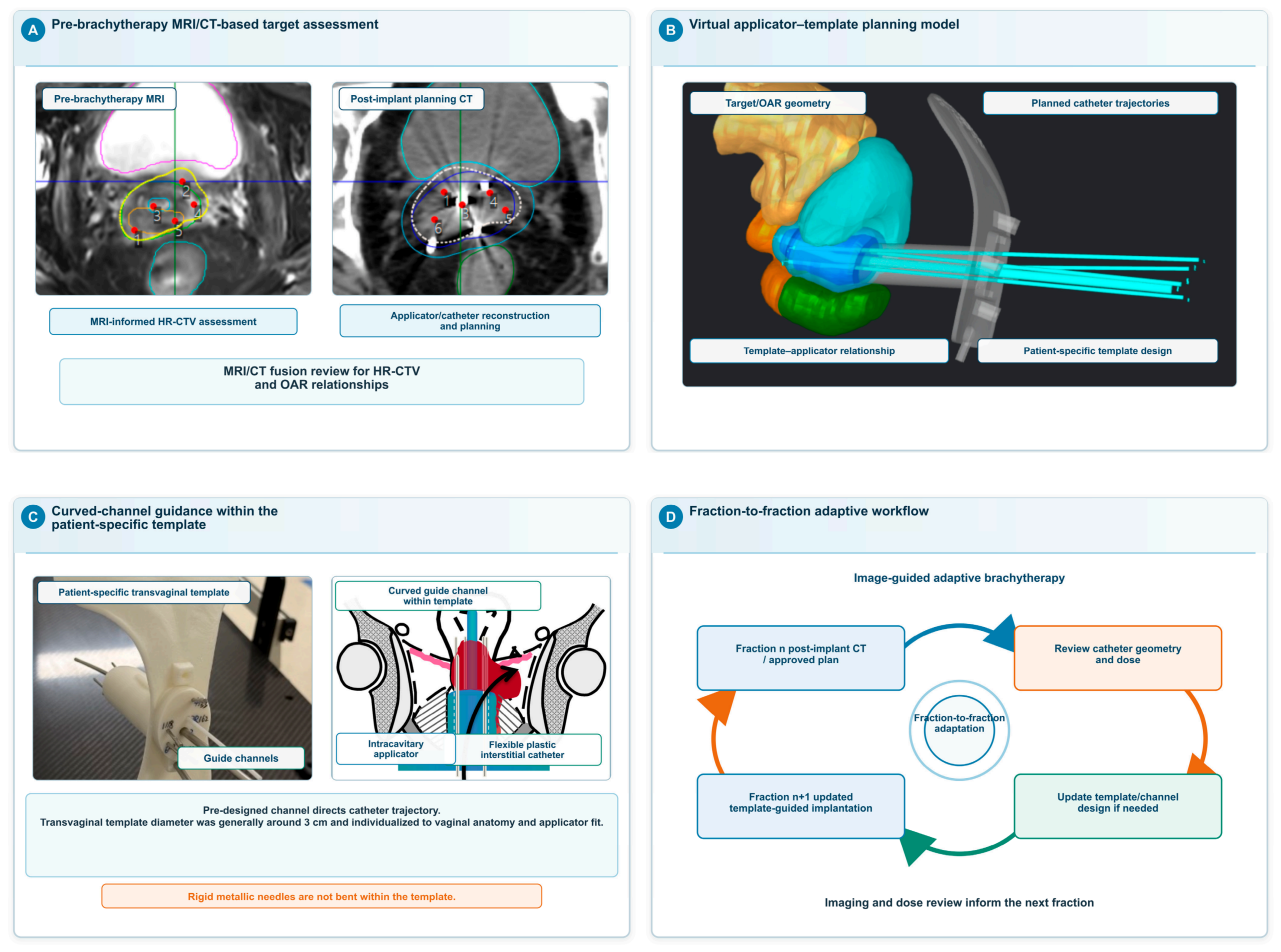
Patient-specific template-guided adaptive brachytherapy workflow and curved-channel guidance concept. (**A**) Pre-brachytherapy MRI and CT were reviewed to define residual tumor extent, HR-CTV, and OAR relationships. (**B**) A virtual applicator–template model was generated to integrate target geometry, OAR proximity, the intracavitary applicator, and planned guide-channel trajectories. (**C**) The patient-specific transvaginal template incorporated straight or curved guide channels according to the planned catheter trajectories. Curved-channel guidance was achieved using pre-designed curved or oblique guide channels within the template and flexible plastic interstitial catheters. The transvaginal template diameter was generally around 3 cm and was individualized to vaginal anatomy and applicator fit. (**D**) For subsequent fractions, post-implant CT, catheter geometry, dose distribution, and the approved treatment plan were reviewed to update catheter trajectories and template/channel design when needed. The MRI and CT panels illustrate complementary imaging roles within the workflow and should not be interpreted as geometrically identical acquisitions without registration review.

All brachytherapy fractions were performed using patient-specific 3D-printed template guidance. A total of 555 template-guided HDR brachytherapy fractions were delivered in the 120-patient cohort. The median number of brachytherapy fractions per patient was 5 (IQR, 4–5; range, 3–5). Template guidance was used at each fraction rather than only at the initial implantation. For subsequent fractions, the most recent post-implant imaging, catheter geometry, approved treatment plan, and dose distribution were reviewed to update the catheter trajectories and template design for the next fraction when needed. This iterative process allowed fraction-to-fraction adaptation of catheter trajectories and template guidance according to tumor regression, applicator geometry, catheter feasibility, and changes in pelvic anatomy. The overall patient-specific template-guided adaptive brachytherapy workflow and curved-channel guidance concept are illustrated in Figure 2.

### 2.4. Template Design, Catheter Trajectory Planning, and Quality Assurance

The technical principles and early clinical implementation of our 3D-printed template and curved-channel catheter system have been described previously [21]. In the present study, this workflow was standardized and evaluated in a consecutive real-world cohort of patients with complex LACC.

The patient-specific template was designed as a catheter-guidance device rather than a generic perineal template. Its primary function was to serve as a physical translation interface between CT/MRI-based pre-planning and intraoperative catheter placement. Template modeling was performed using pre-brachytherapy imaging, the intended intracavitary applicator geometry, residual tumor distribution, HR-CTV, and adjacent OARs. Each template incorporated individualized guide channels specifying catheter entry position, initial direction, intended insertion depth, and spatial relationship to the intracavitary applicator. The template was designed around the intended intracavitary applicator and served as an applicator-associated guidance platform. The applicator provided the central intracavitary component, whereas the printed template provided patient-specific interstitial guide channels surrounding or adjacent to the applicator according to tumor geometry. The relative position between the applicator and the template was fixed during design and verified before implantation, allowing the planned spatial relationship between the intracavitary source path and interstitial catheters to be reproduced during treatment.

The diameter and shape of the transvaginal template component were individualized according to vaginal anatomy, the intended intracavitary applicator size, and the need to maintain stable applicator–template coupling without excessive pressure on the vaginal wall. Guide-channel diameters were matched to the outer diameter of the selected catheter or needle.

Catheter trajectories were planned according to residual tumor geometry, parametrial or vaginal extension, uterine position, and proximity to the bladder, rectum, sigmoid colon, and bowel. Straight channels were designed when a linear trajectory could adequately cover the target while respecting OAR constraints. Curved or oblique channels were designed when lateral, eccentric, or anatomically constrained target regions required a non-linear or non-standard access direction. In these cases, flexible plastic interstitial catheters were used to follow the individualized channel geometry and extend catheter access toward difficult-to-reach target regions.

Curved-channel guidance did not imply that a rigid metallic needle was physically bent within the printed template. Instead, the patient-specific template defined the catheter entry point, initial direction, insertion depth, and applicator-related geometry. For curved-channel or oblique trajectories, flexible plastic interstitial catheters were advanced through pre-designed curved or oblique guide channels within the patient-specific template and followed the planned access geometry toward the target region. In this setting, the printed template functioned as a reproducible geometric guide for the proximal catheter path, while the flexibility of the plastic catheter allowed adaptation to the planned trajectory within soft tissue. All curved-channel designs were reviewed before printing to ensure catheter passability, avoid excessive angulation, and maintain an unobstructed afterloading source pathway.

Several design and safety checks were applied before printing. Catheter trajectories were reviewed to avoid direct traversal of OARs and to minimize the risk of vascular or visceral injury. Potential channel collision, excessive angulation, insufficient spacing, and afterloading pathway obstruction were assessed during virtual modeling. Channel numbering, planned insertion depth, and intended catheter type were documented before implantation to facilitate reproducible intraoperative execution.

Templates were manufactured using medical-grade polylactic acid with a printing resolution of approximately 0.2 mm. After printing, each template was inspected for structural integrity, channel patency, labeling accuracy, and compatibility with the intended applicator. The template was sterilized using low-temperature hydrogen peroxide plasma, and channel patency and mechanical fit were rechecked after sterilization.

Template printing generally required approximately 5–10 h depending on model complexity, channel number, and template geometry. Before sterilization, the template surface was cleaned and wiped with 75% ethanol. Low-temperature hydrogen peroxide plasma sterilization was performed according to the manufacturer’s sterilizer workflow; a full sterilization cycle generally required approximately 50–55 min at 50 °C ± 5 °C. Channel patency and mechanical fit were rechecked after sterilization.

Quality assurance was performed at multiple stages. Before implantation, the radiation oncologist and medical physicist jointly reviewed image registration, target and OAR contours, catheter trajectories, and anticipated dosimetric feasibility. During implantation, the applicator and template were positioned according to the predefined geometry, and catheters were inserted through the corresponding numbered channels to the planned depths. Dummy source or catheter transit checks were performed to confirm unobstructed afterloading pathways before treatment delivery. After implantation, CT-based catheter reconstruction, applicator verification, OAR evaluation, and dose optimization were reviewed by the treating physician and medical physicist. Final treatment plans were approved only after confirming HR-CTV coverage, OAR dose constraints, catheter reconstruction accuracy, and dwell-source pathway integrity.

This study assessed implementation fidelity using clinically available quality indicators, including successful catheter reconstruction on post-implant CT, absence of afterloading source-pathway obstruction, absence of clinically significant applicator displacement, and physician–physicist approval of catheter geometry before treatment delivery. Quantitative planned-versus-achieved catheter trajectory deviation, angular deviation, and deformable accumulated dose were not systematically measured.

### 2.5. Dosimetric, Procedural, and Clinical Outcome Assessment

Treatment planning was performed using Oncentra Brachy version 4.6, software build 4.6.3.12 (Nucletron B.V./Elekta, Veenendaal, The Netherlands), with inverse optimization followed by manual adjustment when needed. Dose prescription followed contemporary image-guided adaptive brachytherapy recommendations, aiming for a cumulative HR-CTV D90 of ≥85–90 Gy EQD2 (α/β = 10), while respecting institutional OAR constraints of ≤75 Gy for the rectum, ≤80 Gy for the bladder, and ≤70 Gy for the sigmoid colon and bowel (α/β = 3) [4,5,30]. Cumulative EBRT and brachytherapy doses were converted to EQD2 using an α/β ratio of 10 Gy for tumor and 3 Gy for OARs.

Recorded dosimetric parameters included HR-CTV volume, HR-CTV D90, V100, conformity index (COIN), dose homogeneity index (DHI), and D2cc values for the bladder, rectum, sigmoid colon, and bowel. Procedural deliverability was assessed using applicator-and-catheter placement time, number of implanted catheter/needle channels, insertion-related bleeding, afterloading pathway obstruction, and clinically significant applicator displacement.

Applicator-and-catheter placement time was defined as the interval from the start of intracavitary applicator insertion after completion of patient preparation, disinfection, and anesthesia to completion of template positioning and interstitial catheter or needle placement. This metric excluded anesthesia induction, patient positioning, imaging acquisition, contouring, treatment planning, irradiation delivery, and post-procedure recovery. It was recorded to assess procedural efficiency and the practical deliverability of the patient-specific template-guided workflow in routine clinical practice.

Clinical outcomes included overall survival (OS), progression-free survival (PFS), local recurrence-free survival (LRFS), regional recurrence-free survival (RRFS), and distant metastasis-free survival (DMFS). Treatment-related toxicity was graded according to the Common Terminology Criteria for Adverse Events, version 5.0. Additional dosimetric parameters and group balance assessments are provided in Supplementary Tables S1 and S2, and detailed dosimetric index calculations are described in the Supplementary Methods.

### 2.6. Statistical Analysis

Continuous variables are presented as medians with interquartile ranges (IQRs) or ranges, as appropriate. Categorical variables are summarized as frequencies and percentages. Group-wise baseline, procedural, and dosimetric data according to catheter-guidance strategy were summarized descriptively. No formal hypothesis testing or *p*-values were used for these group-wise comparisons because straight-channel guidance and curved-channel guidance were selected according to case complexity rather than assigned as balanced treatment groups. Standardized mean differences were used in Supplementary Table S1 to describe baseline and dosimetric imbalance.

Overall survival (OS), progression-free survival (PFS), local recurrence-free survival (LRFS), regional recurrence-free survival (RRFS), and distant metastasis-free survival (DMFS) were estimated using the Kaplan–Meier method. Time-to-event outcomes were calculated from the start of treatment to the date of the relevant event or last follow-up. Median follow-up was estimated using the reverse Kaplan–Meier method. Survival estimates were reported only when the available follow-up supported estimation at the corresponding time point.

Cox proportional hazards regression analyses were performed on an exploratory basis to describe associations between selected clinical or dosimetric variables and survival outcomes. Candidate variables were selected based on clinical relevance and univariable findings. Given the retrospective design, limited event number, baseline imbalance between catheter-guidance strategies, and potential confounding by indication, multivariable models were interpreted as hypothesis-generating rather than confirmatory. No causal inference was made regarding catheter-guidance strategy.

Restricted cubic spline (RCS) models were fitted within the Cox regression framework to explore potential non-linear associations between HR-CTV D90 and survival outcomes. In addition, data-driven stratification and time-dependent receiver operating characteristic analyses were performed as exploratory assessments of dose–outcome patterns. These analyses were not intended to define or validate a definitive clinical dose threshold.

All statistical analyses were performed using R software (version 4.5.1; R Foundation for Statistical Computing, Vienna, Austria). Survival modeling, restricted cubic spline analyses, data-driven stratification, and time-dependent ROC analyses were performed on an exploratory basis and interpreted in the context of clinical plausibility, follow-up maturity, event number, and potential treatment-selection bias.

## 3. Results

### 3.1. Patient Characteristics and Disease Complexity

A total of 120 consecutive patients with FIGO 2018 stage IB3–IVA cervical cancer were included in the final analysis. Baseline characteristics are summarized in Table 1. The median age was 58.1 years, and the cohort was enriched for advanced and anatomically complex disease, with 102 patients (85.0%) presenting with FIGO stage IIIA–IVA tumors. The median tumor size was 5.4 cm, and 44 patients (36.7%) had tumors larger than 6 cm. Squamous cell carcinoma was the predominant histology.

At brachytherapy qualification, residual disease and parametrial or vaginal involvement were reassessed using pre-brachytherapy MRI and clinical examination. Because quantitative residual GTV regression from baseline was not systematically recorded, HR-CTV volume at brachytherapy planning was used as the main imaging-based measure of residual target burden. The median HR-CTV volume at brachytherapy was 55.9 cm^3^, indicating a substantial residual target volume despite prior chemoradiotherapy.

Within the patient-specific template-guided workflow, catheter-guidance strategy was individualized according to residual tumor geometry and pelvic anatomy. Curved-channel guidance was preferentially used in patients with greater disease complexity. Compared with patients treated using predominantly straight-channel guidance, those requiring curved-channel guidance had a higher proportion of FIGO stage IIIA–IVA disease and larger tumors. This distribution reflects clinical selection of curved-channel trajectories for more difficult implantation scenarios rather than balanced allocation between treatment groups.

### 3.2. Clinical Implementation and Workflow Deliverability

Workflow deliverability is summarized in Table 2. In total, 555 template-guided HDR brachytherapy fractions were delivered in 120 patients. The median number of brachytherapy fractions per patient was 5 (IQR, 4–5; range, 3–5), including 77 patients treated with 5 fractions, 41 treated with 4 fractions, and 2 treated with 3 fractions. Template guidance was used for every fraction. Between fractions, the most recent post-implant imaging and approved treatment plan were used to inform subsequent catheter trajectory and template updates when needed.

HDR brachytherapy was generally delivered twice weekly. The number of fractions was individualized according to residual disease extent, target coverage, and OAR constraints, with most patients receiving four or five fractions.

The workflow was procedurally efficient in routine practice. The median applicator-and-catheter placement time was 4.21 min per fraction, reflecting the interval required to place the intracavitary applicator, position the patient-specific template, and insert the planned interstitial catheters or needles after completion of patient preparation, disinfection, and anesthesia. The median number of implanted channels was 7.25 per fraction. Insertion-related complications were uncommon, with minor bleeding observed in 13 patients (10.8%) and major bleeding in 2 patients (1.7%). Across all template-guided fractions, no afterloading source-pathway obstruction or clinically significant applicator displacement was observed.

For the first brachytherapy fraction, image review, contour review, catheter trajectory planning, and template modeling usually required approximately 120–180 min, depending on tumor complexity and the number of planned channels. Three-dimensional printing, post-printing inspection, and sterilization were completed before implantation according to the institutional workflow. These pre-procedure steps were not included in the applicator-and-catheter placement time reported in Table 2.

In routine practice, template design was reviewed on a weekly basis. A new or updated template was generally prepared for each treatment week according to the most recent post-implant CT, approved plan, catheter geometry, and tumor/anatomical changes. When two fractions were delivered within the same week and the applicator–template geometry remained appropriate, the same weekly template design was used for both fractions. Thus, all patients underwent planned weekly template review and update, whereas intra-week reprinting was not routinely required.

### 3.3. Dosimetric Performance of the Template-Guided Adaptive Workflow

Dosimetric outcomes are summarized in Table 3. Across the whole cohort, the median HR-CTV volume was 55.9 cm^3^, and the median cumulative HR-CTV D90 was 92.9 Gy EQD2, indicating that guideline-consistent target coverage was achievable in this clinically complex population. Median OAR D2cc values remained within contemporary institutional and guideline-consistent constraints, including 72.5 Gy EQD2 for rectum, 76.2 Gy EQD2 for bladder, 64.9 Gy EQD2 for sigmoid colon, and 63.0 Gy EQD2 for bowel.

The median COIN was 0.7, suggesting acceptable conformity of the prescription isodose to the residual target in this anatomically complex cohort. The median DHI was 0.5, reflecting the expected dose heterogeneity of hybrid intracavitary/interstitial HDR brachytherapy, in which high-dose regions around intracavitary and interstitial source paths are inherent to the technique. V100 values were therefore interpreted together with HR-CTV D90, COIN, DHI, and OAR D2cc rather than as isolated indicators of plan quality.

Patients treated with curved-channel guidance had larger HR-CTV volumes than those treated with predominantly straight-channel guidance, consistent with preferential use of curved-channel trajectories in more extensive or anatomically constrained tumors. Despite this greater baseline complexity, target coverage and OAR doses remained clinically acceptable. These results support the dosimetric feasibility of using individualized catheter-guidance strategies within the same patient-specific template-guided adaptive brachytherapy workflow.

### 3.4. Curved-Channel Guidance for Complex Target Geometry

Curved-channel guidance was used as an individualized catheter-guidance strategy within the same patient-specific template-guided adaptive workflow. It was preferentially selected for patients with more extensive residual disease, lateral or parametrial target extension, unfavorable pelvic anatomy, or catheter trajectories that were unlikely to be adequately achieved using purely straight metallic needle paths.

Compared with patients treated using predominantly straight-channel guidance, patients requiring curved-channel guidance had greater baseline disease complexity, including a higher proportion of FIGO stage IIIA–IVA disease, larger tumors, and larger HR-CTV volumes. This imbalance reflects the clinical role of curved-channel guidance as an adaptive solution for anatomically challenging cases rather than a balanced comparative treatment allocation. Therefore, outcome differences between catheter-guidance strategies were interpreted descriptively and were not used to infer superiority of one guidance strategy over another.

Despite the greater disease complexity in the curved-channel guidance group, guideline-consistent HR-CTV coverage and acceptable OAR doses were maintained. These findings suggest that curved-channel catheter guidance using flexible plastic interstitial catheters can expand the spatial reach of hybrid IC/IS brachytherapy within a patient-specific template-guided workflow, particularly for eccentric or anatomically constrained target regions.

### 3.5. Early Clinical Outcomes and Toxicity Within the Available Follow-Up

**Figure 3:**
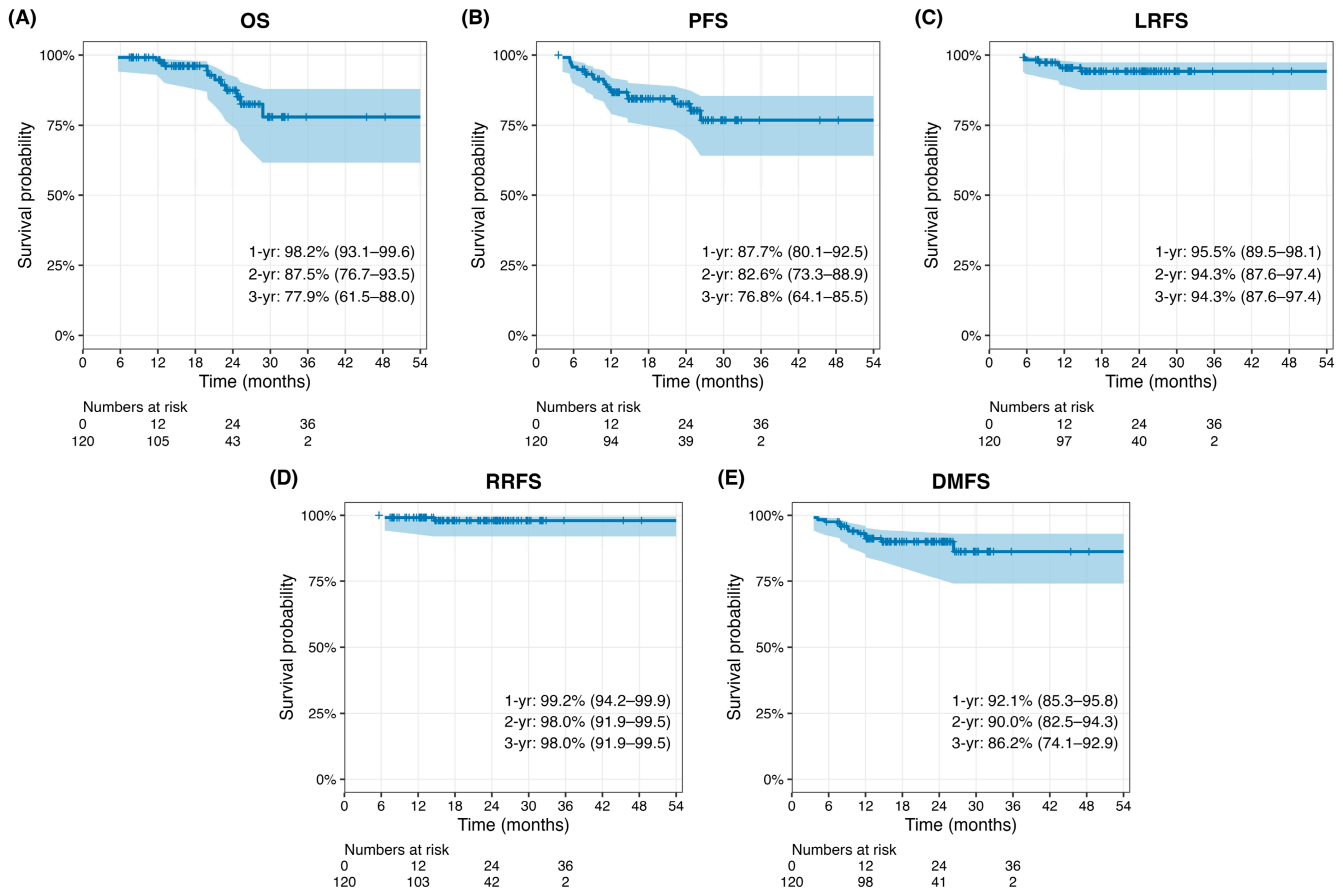
Kaplan–Meier estimates of early clinical outcomes within the available follow-up period. Kaplan–Meier curves for (**A**) overall survival (OS), (**B**) progression-free survival (PFS), (**C**) local recurrence-free survival (LRFS), (**D**) regional recurrence-free survival (RRFS), and (**E**) distant metastasis-free survival (DMFS). Outcome estimates should be interpreted in the context of the current follow-up maturity and the number of patients remaining at risk at later time points. Numbers at risk are shown below each panel.

At a median follow-up of 20.1 months, early-to-intermediate-term clinical outcomes were favorable within the available follow-up period (Table 4 and Figure 3). The estimated 3-year OS, PFS, LRFS, RRFS, and DMFS rates were 77.9%, 76.8%, 94.3%, 98.0%, and 86.2%, respectively. Local and regional pelvic control were particularly favorable, as reflected by the high LRFS and RRFS rates. These estimates should be interpreted in the context of the current follow-up maturity and the number of patients remaining at risk at later time points.

Treatment-related toxicity is summarized in Table 5. Acute grade 1–2 gastrointestinal and genitourinary toxicities occurred in 56 patients (46.7%) and 28 patients (23.3%), respectively. Acute grade ≥ 3 toxicity was uncommon, with one grade ≥ 3 genitourinary event and no acute grade ≥ 3 gastrointestinal events. Late grade ≥ 3 gastrointestinal and genitourinary toxicities occurred in 3 patients (2.5%) and 2 patients (1.7%), respectively. No grade 4–5 toxicity or treatment-related death was observed. Although longer follow-up is required for mature late-toxicity assessment, the current data support an acceptable early safety profile for this patient-specific template-guided adaptive brachytherapy workflow.

### 3.6. Exploratory Dose–Outcome Analyses

Exploratory analyses were performed to describe possible associations between clinical or dosimetric variables and survival outcomes. Disease-burden variables, including FIGO stage and tumor size, showed the most clinically consistent associations with adverse outcomes. Associations involving HR-CTV D90 were less stable across models and were interpreted cautiously because delivered dose, catheter-guidance strategy, and baseline tumor complexity were closely interrelated in routine practice. These analyses were hypothesis-generating, were not intended to validate a dose threshold, and remained vulnerable to confounding by indication, limited follow-up, and small event numbers.

Additional exploratory analyses, including data-driven Kaplan–Meier stratification, Cox regression, restricted cubic spline modeling, and time-dependent ROC analysis, are provided in the Supplementary Materials. All supplementary dose–outcome analyses were considered hypothesis-generating and not threshold-validating.

## 4. Discussion

In this real-world single-center cohort, image-guided adaptive brachytherapy using patient-specific 3D-printed templates was feasible for patients with complex LACC. The cohort was enriched for advanced and anatomically challenging disease, with 85.0% of patients presenting with FIGO stage IIIA–IVA tumors and more than one-third having tumors larger than 6 cm. Across 555 template-guided HDR brachytherapy fractions, patient-specific template guidance was used at every fraction, with catheter trajectories and template design updated between fractions when needed based on the most recent post-implant imaging and treatment plan. This fraction-level implementation achieved guideline-consistent HR-CTV coverage, acceptable OAR doses, efficient applicator-and-catheter placement, favorable early pelvic control, and low severe toxicity within the available follow-up period.

The present study should be interpreted within the established framework of image-guided adaptive brachytherapy for cervical cancer. Landmark studies such as RetroEMBRACE and EMBRACE-I demonstrated that MRI- or CT-based adaptive brachytherapy, volumetric target definition, and DVH-based dose optimization improve pelvic control while maintaining acceptable morbidity [8,9,10,31]. Subsequent dose-planning recommendations have emphasized cumulative HR-CTV D90 targets in the range of approximately 85–90 Gy EQD2, balanced against OAR constraints [11,30]. Our study was not designed to redefine these dose objectives. Instead, its main contribution lies in implementation: it demonstrates that contemporary IGABT principles can be translated into a reproducible patient-specific template-guided workflow for bulky, asymmetric, or anatomically constrained cervical tumors in routine practice.

**Figure 4:**
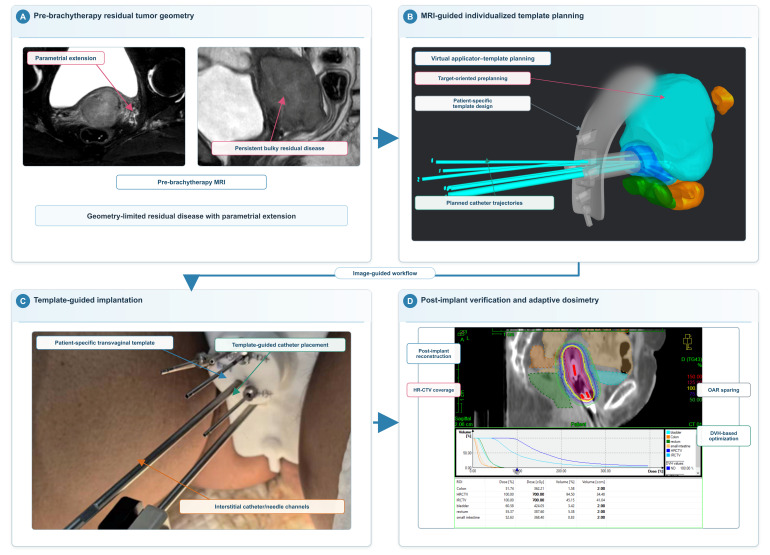
Representative case illustrating image-based individualized planning and template-guided implementation. (**A**) Pre-brachytherapy MRI after chemoradiotherapy showed persistent bulky residual cervical disease with parametrial extension, representing a geometry-limited target configuration for standard intracavitary brachytherapy alone. (**B**) MRI-guided individualized preplanning was used to generate a virtual applicator–template model and planned catheter trajectories according to residual target geometry and OAR relationships. (**C**) A patient-specific transvaginal template was fabricated and used for template-guided catheter placement. (**D**) Post-implant sagittal reconstruction and DVH-based plan review were used to assess applicator/catheter geometry, HR-CTV coverage, OAR sparing, and adaptive dosimetry.

A central feature of this workflow is the role of the 3D-printed template as a physical translation interface between image-based pre-planning and intraoperative catheter placement. In complex LACC, the main challenge is not only target delineation or dose optimization but also the accurate and reproducible execution of the intended catheter geometry. By incorporating individualized guide channels, planned insertion depths, channel numbering, applicator relationships, and post-printing quality assurance, the patient-specific template allowed CT/MRI-based trajectory planning to be converted into a practical implantation procedure. The use of template guidance in all 555 fractions highlights that this approach was not limited to selected proof-of-concept cases but was incorporated into routine fraction-level adaptive brachytherapy. A representative case illustrating how image-based individualized planning was translated into template-guided implantation and post-implant adaptive dosimetry is shown in Figure 4.

Curved-channel guidance should be understood as an adaptive catheter-guidance strategy within the same patient-specific template-guided workflow rather than as a competing treatment group. In our cohort, curved-channel guidance was preferentially used in patients with greater disease complexity, including more advanced stage, larger tumors, and larger HR-CTV volumes. This imbalance reflects clinical decision-making: curved or oblique catheter trajectories were selected when eccentric, lateral, parametrial, vaginal, or anatomically constrained target extensions were unlikely to be adequately reached by purely straight metallic needle paths. Despite this unfavorable baseline profile, clinically acceptable target coverage and OAR doses were maintained. These findings suggest that curved-channel guidance using flexible plastic interstitial catheters may expand the spatial reach of hybrid IC/IS brachytherapy for difficult target geometries, but they should not be interpreted as evidence of superiority over straight-channel guidance.

The procedural findings are also clinically relevant. Patient-specific template-guided and hybrid IC/IS workflows are often perceived as technically demanding, time-consuming, or difficult to disseminate. In this study, the median applicator-and-catheter placement time was short, and no afterloading source-pathway obstruction or clinically significant applicator displacement was observed across all template-guided fractions. This suggests that shifting much of the geometric decision-making to the pre-implant planning stage may improve procedural efficiency during implantation, even when individualized catheter trajectories are used. The low rate of major insertion-related bleeding further supports the practical safety of this workflow when combined with systematic design review, channel patency checks, post-implant imaging, and physician–physicist quality assurance.

The early clinical outcomes and toxicity profile were encouraging but should be interpreted cautiously. Local and regional control were favorable within the available follow-up period, and severe late gastrointestinal and genitourinary toxicity rates were low. These findings are consistent with the expectation that guideline-consistent target coverage and OAR sparing can support a favorable therapeutic ratio. However, the median follow-up of 20.1 months remains insufficient for a mature assessment of late morbidity, especially for gastrointestinal, genitourinary, and vaginal sequelae that may develop over longer intervals. Therefore, the toxicity data should be viewed as an early safety profile rather than a definitive estimate of long-term morbidity.

The dose–outcome analyses in this study were exploratory. Although HR-CTV D90 showed possible associations with survival outcomes in stratified, spline-based, and time-dependent ROC analyses, these findings were vulnerable to confounding by indication. In routine practice, larger and more technically difficult tumors were more likely to require intensified catheter guidance, curved-channel trajectories, and dose escalation attempts. As a result, delivered dose, catheter-guidance strategy, and baseline disease complexity were closely interrelated. The apparent non-linear pattern around 90–93 Gy EQD2 should therefore be regarded as hypothesis-generating rather than as validation of a new clinical threshold. The results support the clinical relevance of maintaining adequate target coverage but do not justify changing established dose objectives.

Several limitations should be acknowledged. First, this retrospective single-center study has limited generalizability. Second, although the cohort included 120 consecutive patients and 555 template-guided fractions, follow-up remains relatively short for definitive survival and late-toxicity conclusions. Third, straight-channel guidance and curved-channel guidance were selected according to tumor geometry and pelvic anatomy rather than assigned in a balanced or randomized manner; comparisons between catheter-guidance strategies are therefore descriptive. Fourth, the limited number of survival events reduced the stability of multivariable and dose–outcome modeling. Fifth, quantitative tumor regression metrics, including paired baseline-to-brachytherapy GTV regression and parametrial infiltration scores, were not systematically captured. Finally, although the workflow incorporated multi-stage quality assurance, planned-versus-achieved catheter positional deviation, angular deviation, inter-fraction geometric variation, and deformable dose accumulation were not systematically measured. Accordingly, the workflow supports clinical feasibility and implementation but does not provide formal geometric-accuracy validation of the template system. Future prospective studies should include longer follow-up, prospective workflow registries, multi-institutional validation, predefined catheter-deviation metrics, angular error analysis, deformable dose accumulation, and further standardization of template design and quality assurance.

Despite these limitations, the present study provides practical evidence supporting the clinical implementation of patient-specific 3D-printed template-guided adaptive brachytherapy for complex LACC. By linking CT/MRI-based pre-planning, individualized catheter trajectory design, fraction-to-fraction adaptation, and structured quality assurance, this workflow may help bridge the gap between advanced image-guided planning and reproducible implantation in routine brachytherapy practice.

## 5. Conclusions

This real-world implementation study supports the feasibility of image-guided adaptive brachytherapy using patient-specific 3D-printed templates for complex LACC. Across 555 template-guided fractions, the workflow enabled individualized straight-channel and curved-channel catheter guidance, fraction-to-fraction adaptation, guideline-consistent target coverage, acceptable OAR doses, efficient applicator-and-catheter placement, and low severe toxicity within the available follow-up period. Curved-channel guidance should be interpreted as an adaptive strategy for anatomically challenging target geometry rather than as a superior alternative to straight-channel guidance. Exploratory dose–outcome analyses support the clinical relevance of adequate HR-CTV coverage but do not validate a definitive dose threshold. Longer follow-up, prospective validation, and quantitative implantation-accuracy assessment are warranted.

## Figures and Tables

**Table 1:** Baseline patient characteristics and disease complexity according to catheter-guidance strategy within the patient-specific template-guided workflow.

Characteristic	All Patients (*n* = 120)	Straight-Channel Guidance	Curved-Channel Guidance
Age (years), median (Q1–Q3)	58.1 (47.7–62.3)	57.9 (49.3–62.3)	58.1 (46.2–61.9)
IB3–IIB	18 (15.0%)	16 (26.7%)	2 (3.3%)
IIIA–IVA	102 (85.0%)	44 (73.3%)	58 (96.7%)
Tumor size (cm), median (Q1–Q3)	5.4 (4.4–6.4)	5.3 (4.1–6.2)	5.9 (4.8–7.0)
≤6 cm	76 (63.3%)	43 (71.7%)	33 (55.0%)
>6 cm	44 (36.7%)	17 (28.3%)	27 (45.0%)
SCC	113 (94.2%)	57 (95.0%)	56 (93.3%)
Non-SCC	7 (5.8%)	3 (5.0%)	4 (6.7%)
Overall treatment time (weeks), median (Q1–Q3)	8.3 (7.1–9.1)	8.2 (7.9–9.2)	8.3 (6.9–9.1)
Concurrent chemotherapy, *n* (%)	120 (100.0%)	60 (100.0%)	60 (100.0%)

Values are presented as median (Q1–Q3) or number (percentage). Straight-channel guidance and curved-channel guidance describe catheter-guidance strategies used within the same patient-specific 3D-printed template-guided adaptive brachytherapy workflow. These categories were selected according to residual tumor geometry and pelvic anatomy and were not intended as balanced comparative treatment groups. Abbreviations: SCC = squamous cell carcinoma. No formal statistical comparisons or *p*-values are provided because catheter-guidance strategy was selected according to case complexity rather than assigned as a balanced treatment group; group-wise values are presented descriptively.

**Table 2:** Workflow deliverability and applicator/catheter placement characteristics of the patient-specific 3D-printed template-guided adaptive brachytherapy workflow.

Parameter	Value
Number of patients	120
Total template-guided brachytherapy fractions	555
Brachytherapy fractions per patient	5 (4–5; range, 3–5)
Patients treated with 3 fractions	2/120 (1.7%)
Patients treated with 4 fractions	41/120 (34.2%)
Patients treated with 5 fractions	77/120 (64.2%)
Template-guided fractions	555/555 (100%)
Applicator-and-catheter placement time, min per fraction	4.21 (3.54–4.91)
Number of implanted catheter/needle channels per fraction	7.25 (5.46–8.96)
Minor insertion-related bleeding	13/120 (10.8%)
Major insertion-related bleeding	2/120 (1.7%)
Afterloading source-pathway obstruction	0/555 (0%)
Clinically significant applicator displacement	0/555 (0%)

Values are presented as median (IQR; range where indicated) or *n*/*N* (%). Applicator-and-catheter placement time was measured from the start of intracavitary applicator insertion after completion of patient preparation, disinfection, and anesthesia to completion of template positioning and interstitial catheter/needle placement. This metric excluded anesthesia induction, patient positioning, imaging acquisition, contouring, treatment planning, irradiation delivery, and post-procedure recovery. Template-guided fractions refer to fractions in which patient-specific 3D-printed template guidance was used for applicator and catheter placement. For subsequent fractions, catheter trajectories and template design were updated based on the most recent post-implant imaging and approved treatment plan when clinically indicated.

**Table 3:** Dosimetric performance of patient-specific template-guided adaptive brachytherapy according to catheter-guidance strategy.

Parameter	All Patients (*n* = 120)	Straight-Channel Guidance	Curved-Channel Guidance
HR-CTV volume (cm^3^)	55.9 (45.2–70.6)	46.6 (36.9–55.8)	65.0 (55.3–77.1)
HR-CTV D90 (Gy, α/β = 10)	92.9 (89.2–95.5)	89.9 (89.2–95.5)	93.8 (89.2–99.1)
V100 (%)	46.2 (37.6–55.0)	41.0 (31.1–50.6)	49.6 (44.0–58.8)
COIN	0.7 (0.6–0.8)	0.7 (0.7–0.8)	0.7 (0.6–0.8)
DHI	0.5 (0.4–0.5)	0.5 (0.4–0.5)	0.5 (0.4–0.5)
EBRT EQD2 (Gy, α/β = 10)	49.6 (44.2–49.6)	49.6 (44.2–49.6)	49.6 (44.2–49.6)
Brachytherapy EQD2 (Gy, α/β = 10)	45.9 (39.7–49.6)	45.9 (39.7–47.7)	49.6 (39.7–49.6)
Rectum D2cc EQD2 (total, Gy, α/β = 3)	72.5 (68.8–74.9)	72.0 (67.2–74.7)	73.4 (69.9–75.2)
Bladder D2cc EQD2 (total, Gy, α/β = 3)	76.2 (71.5–81.0)	74.5 (70.3–79.5)	77.9 (74.4–82.2)
Sigmoid D2cc EQD2 (total, Gy, α/β = 3)	64.9 (60.1–69.3)	65.1 (59.3–69.2)	64.7 (61.3–70.3)
Bowel D2cc EQD2 (total, Gy, α/β = 3)	63.0 (58.9–67.6)	62.3 (56.6–68.2)	63.6 (59.9–67.4)

All doses are reported as EQD2, using α/β = 10 Gy for tumor and α/β = 3 Gy for organs at risk. Values are presented as median (Q1–Q3). Straight-channel guidance and curved-channel guidance were selected according to target geometry, pelvic anatomy, and anticipated catheter feasibility within the same patient-specific template-guided adaptive workflow. These data are descriptive and should not be interpreted as a causal comparison between guidance strategies. Abbreviations: EQD2 = equivalent dose in 2 Gy fractions; HR-CTV = high-risk clinical target volume; DHI = dose homogeneity index; COIN = conformity index. No formal statistical comparisons or *p*-values are provided because catheter-guidance strategy was selected according to case complexity rather than assigned as a balanced treatment group; group-wise values are presented descriptively.

**Table 4:** Estimated Kaplan–Meier outcomes within the available follow-up period according to catheter-guidance strategy.

Endpoint	All Patients (*n* = 120)			Straight-Channel Guidance			Curved-Channel Guidance		
	1-Year	2-Year	3-Year	1-Year	2-Year	3-Year	1-Year	2-Year	3-Year
OS	98.2% (95.8–100.0%)	87.5% (79.8–95.9%)	77.9% (66.0–92.0%)	98.3% (95.1–100.0%)	90.5% (82.9–98.9%)	84.6% (74.3–96.3%)	97.9% (94.0–100.0%)	73.5% (52.6–100.0%)	—
PFS	87.7% (81.9–94.0%)	82.6% (75.3–90.6%)	76.8% (66.9–88.1%)	88.3% (80.5–96.8%)	84.7% (76.0–94.4%)	78.3% (67.1–91.4%)	87.0% (78.4–96.5%)	74.3% (55.8–98.9%)	—
LRFS	95.5% (91.7–99.4%)	94.3% (89.9–98.9%)	94.3% (89.9–98.9%)	96.4% (91.6–100.0%)	94.4% (88.4–100.0%)	94.4% (88.4–100.0%)	94.6% (88.8–100.0%)	94.6% (88.8–100.0%)	—
RRFS	99.2% (97.5–100.0%)	98.0% (95.2–100.0%)	98.0% (95.2–100.0%)	100.0% (100.0–100.0%)	98.0% (94.3–100.0%)	98.0% (94.3–100.0%)	98.3% (95.1–100.0%)	98.3% (95.1–100.0%)	—
DMFS	92.1% (87.2–97.2%)	90.0% (84.5–95.8%)	86.2% (77.7–95.7%)	91.6% (84.7–98.9%)	89.7% (82.2–97.9%)	85.8% (75.9–97.1%)	92.5% (85.7–99.9%)	90.4% (82.7–98.8%)	—

Survival probabilities at 1, 2, and 3 years are presented with 95% confidence intervals. ‘—’ indicates that the corresponding estimate was not estimable because the available follow-up in that subgroup did not support reliable estimation at that time point; no extrapolation was performed. Catheter-guidance strategy was selected according to case complexity and should not be interpreted as a balanced comparative treatment group. Abbreviations: OS = overall survival; PFS = progression-free survival; LRFS = local recurrence-free survival; RRFS = regional recurrence-free survival; DMFS = distant metastasis-free survival.

**Table 5:** Treatment-related toxicity observed within the current follow-up period.

Toxicity	Acute G1–2 *n* (%)	Acute ≥ G3 *n* (%)	Late G1–2 *n* (%)	Late ≥ G3 *n* (%)
GI (rectum/colon)	56 (46.7%)	0 (0%)	28 (23.3%)	3 (2.5%)
GU (bladder)	28 (23.3%)	1 (0.8%)	8 (6.7%)	2 (1.7%)

Toxicities were graded according to Common Terminology Criteria for Adverse Events (CTCAE), version 5.0. Values are *n* (%). Abbreviations: GI = gastrointestinal; GU = genitourinary; G = grade.

## Data Availability

The datasets generated and/or analyzed during the current study are not publicly available due to institutional data protection policies and patient privacy restrictions. De-identified data may be available from the corresponding author upon reasonable request and subject to institutional approval.

## Supplementary Materials

The following supporting information can be downloaded at: https://www.mdpi.com/article/10.3390/cancers18152399/s1, Figure S1: Descriptive Kaplan–Meier outcome estimates according to catheter-guidance strategy; Figure S2: Exploratory Kaplan–Meier analyses stratified by HR-CTV D90; Figure S3: Exploratory Cox regression analyses for overall survival and progression-free survival; Figure S4: Exploratory restricted cubic spline analyses of the association between HR-CTV D90 and survival outcomes; Figure S5: Time-dependent ROC curves for HR-CTV D90; Table S1: Baseline and dosimetric characteristics according to straight-channel and curved-channel guidance strategies, with standardized mean differences (SMDs); Table S2: Additional dosimetric parameters for image-guided adaptive brachytherapy; Table S3: Exploratory univariable Cox regression analyses for overall survival and progression-free survival; Table S4: Exploratory multivariable Cox regression analyses for overall survival and progression-free survival.

## Abbreviations

3D	three-dimensional
ABS	American Brachytherapy Society
BT	brachytherapy
CI	confidence interval
COIN	conformity index
CT	computed tomography
CTCAE	Common Terminology Criteria for Adverse Events
DHI	dose homogeneity index
DMFS	distant metastasis-free survival
DVH	dose–volume histogram
EBRT	external beam radiotherapy
EQD2	equivalent dose in 2-Gy fractions
FIGO	International Federation of Gynecology and Obstetrics
GEC-ESTRO	Groupe Européen de Curiethérapie–European Society for Radiotherapy and Oncology
GI	gastrointestinal
GTV	gross tumor volume
GU	genitourinary
HDR	high-dose-rate
HR	hazard ratio
HR-CTV	high-risk clinical target volume
IC/IS	intracavitary–interstitial
ICRU	International Commission on Radiation Units and Measurements
IGABT	image-guided adaptive brachytherapy
IMRT	intensity-modulated radiotherapy
IQR	interquartile range
LACC	locally advanced cervical cancer
LRFS	local recurrence-free survival
MRI	magnetic resonance imaging
OAR	organ at risk
OS	overall survival
OTT	overall treatment time
PFS	progression-free survival
PLA	polylactic acid
RCS	restricted cubic spline
ROC	receiver operating characteristic
RRFS	regional recurrence-free survival
SIB	simultaneous integrated boost
VMAT	volumetric modulated arc therapy
