# Supplementary material for: Image-Guided Adaptive Brachytherapy Using Patient-Specific 3D-Printed Templates for Complex Locally Advanced Cervical Cancer: A Real-World Implementation Study
Source: Cancers (Basel). 2026 Jul 25;18(15):2399. doi: 10.3390/cancers18152399 (PMC13465610; doi:10.3390/cancers18152399)
Supplement: Supplementary file 1 [file cancers-18-02399-s001.zip › cancers-4440380-supplementary.pdf]

cancers

MDPI

Supplementary Materials: Image-Guided Adaptive Brachytherapy Using Patient-Specific 3D-Printed Templates for Complex Locally Advanced Cervical Cancer: A Real-World Implementation Study

Yuanjie Cao, Imashi Sandupama Wickramage, Chen Li, Youheng Tan, Wenwen Zhang, Qingsong Pang and Jie Chen

This supplementary file includes additional methodological details, dosimetric and analytical calculations, supplementary tables, and supplementary figure legends supporting the main manuscript.

Supplementary Methods: Dosimetric and Analytical Calculations

The EQD2 was calculated according to the linear–quadratic model:

$$\mathrm{EQD2} = \mathrm{D} \times \frac{\mathrm{d} + \alpha/\beta}{2 + \alpha/\beta}$$

where *D* is the total physical dose, d is the dose per fraction, and α/β is the tissue-specific parameter (10 Gy for tumor tissue in HR-CTV and 3 Gy for normal tissues/organs at risk).

To evaluate conformity and homogeneity, the following indices were calculated.

- **Conformity Index (COIN):**

$$COIN = \left(\frac{TV_{RI}}{TV}\right) \times \left(\frac{TV_{RI}}{V_{RI}}\right)$$

Where TV is the target volume, V_{RI} is the volume of the prescription isodose, and TV_{RI} is the portion of the target volume covered by the prescription isodose.

- **Dose Homogeneity Index (DHI):**

$$DHI = \frac{V_{100} - V_{150}}{V_{100}}$$

Where V_{100} is the target volume receiving 100% of the prescribed dose and V_{150} is the target volume receiving 150% of the prescribed dose.

- **Dose Non-uniformity Ratio (DNR):**

$$DNR = \frac{V_{150}}{V_{100}}$$

with the same definitions for V_{100} and V_{150} as above.

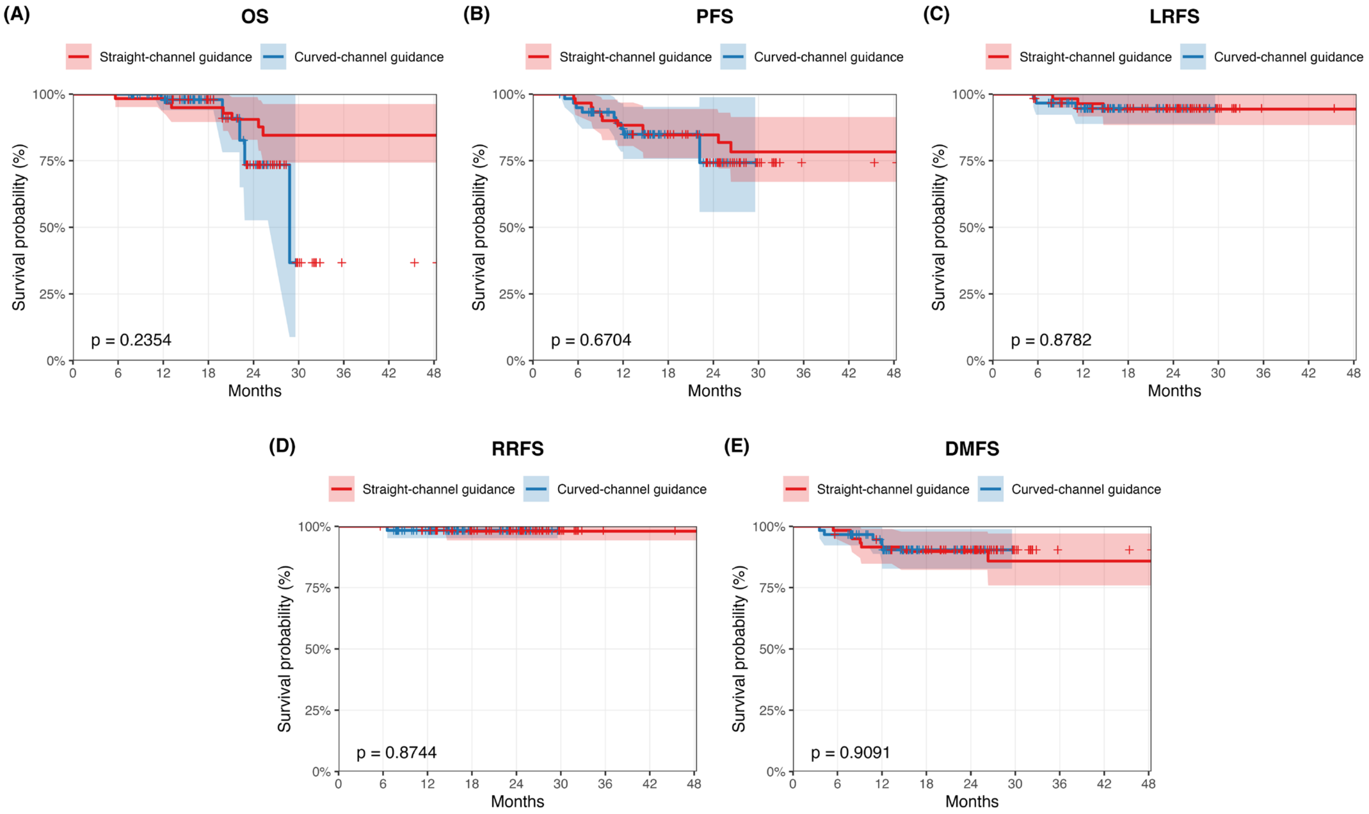

Figure S1. Descriptive Kaplan–Meier outcome estimates according to catheter-guidance strategy. Kaplan–Meier curves comparing patients treated using predominantly straight-channel guidance and those treated using curved-channel guidance. Because catheter-guidance strategy reflected case complexity rather than balanced allocation, these comparisons are descriptive only and should not be interpreted as causal comparative evidence.

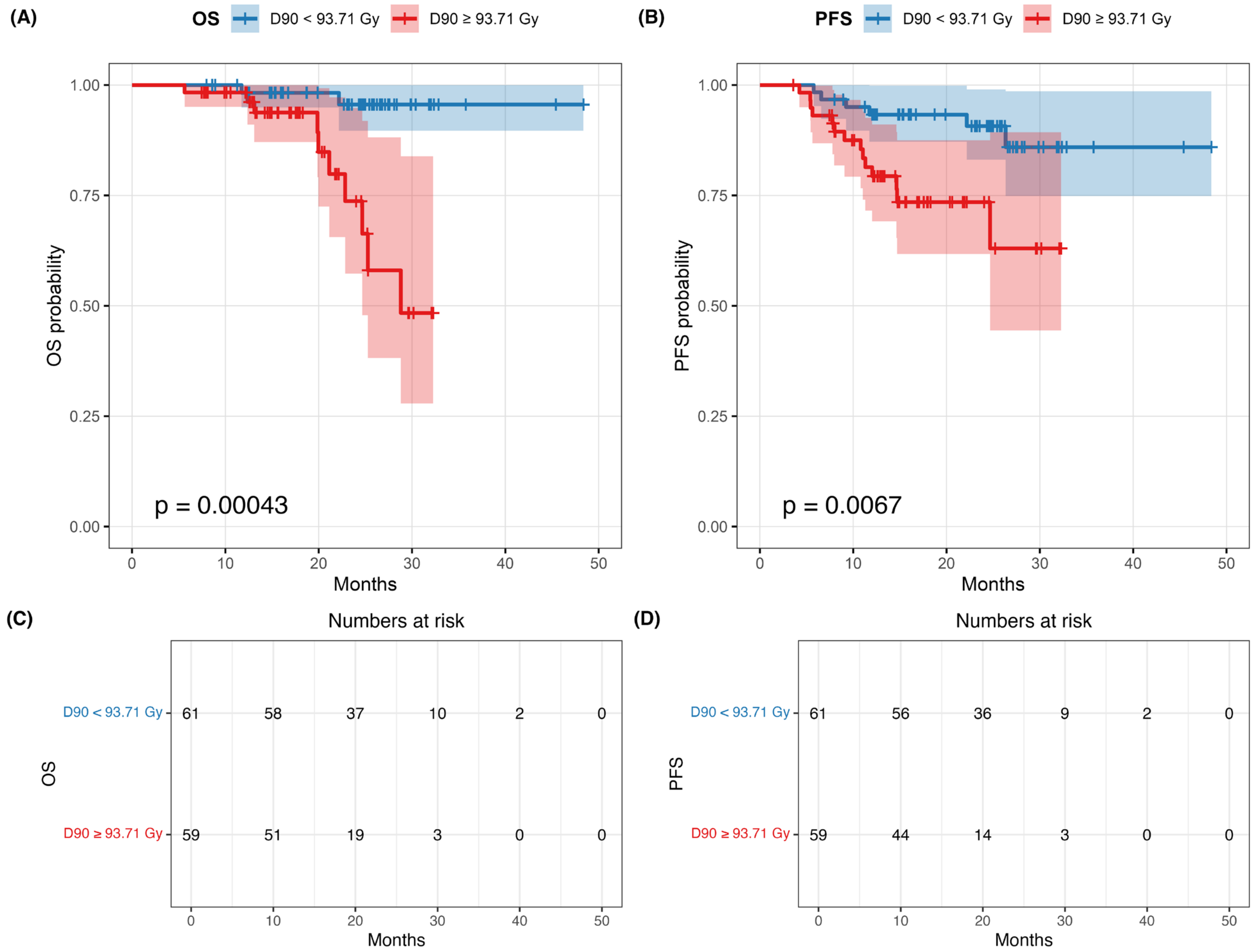

Figure S2. Exploratory Kaplan–Meier analyses stratified by HR-CTV D90. Kaplan–Meier curves for overall survival and progression-free survival according to a data-driven HR-CTV D90 stratification. These analyses were performed on an exploratory basis and should be interpreted cautiously because baseline tumor burden, catheter-guidance strategy, and treatment-selection bias may have influenced the apparent association between dose and outcome. These findings should not be interpreted as validation of a definitive clinical dose threshold. The findings are hypothesis-generating.

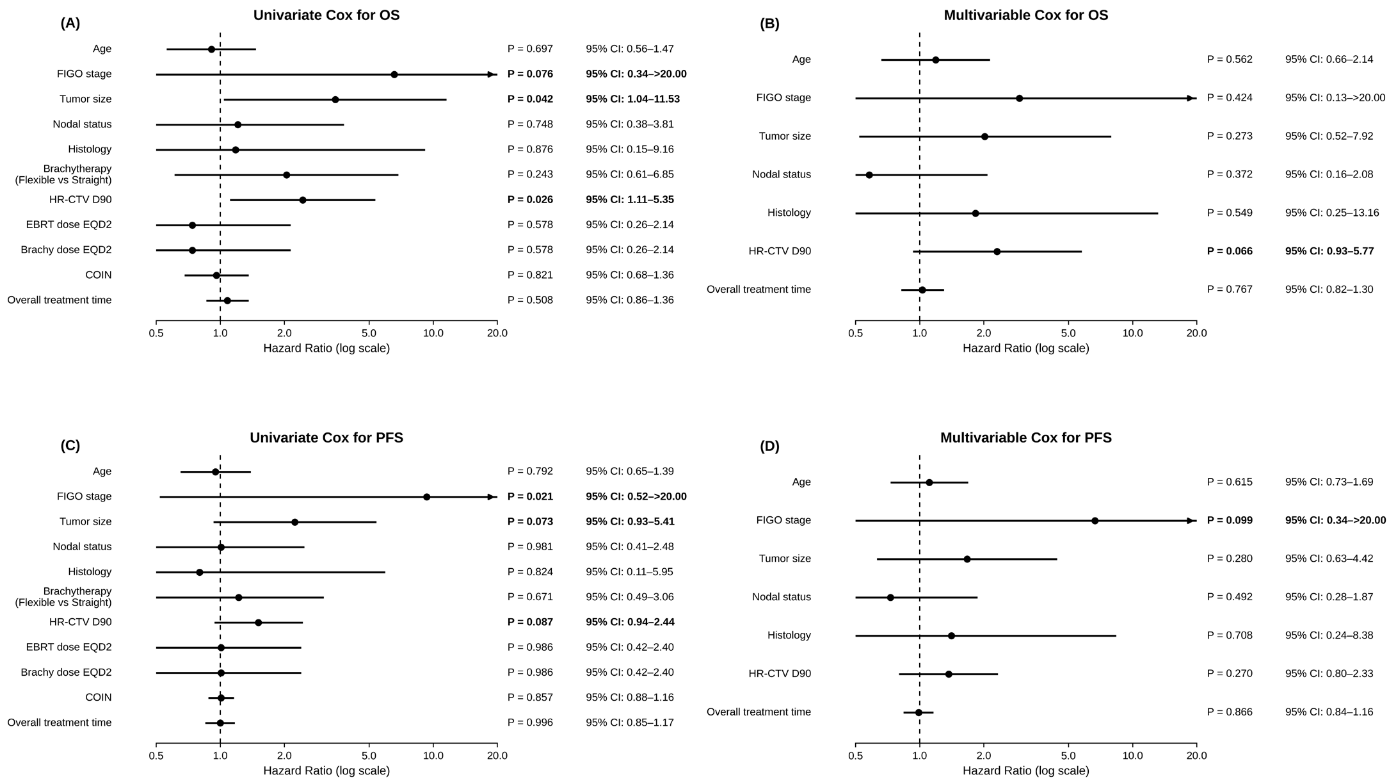

Figure S3. Exploratory Cox regression analyses for overall survival and progression-free survival. Forest plots showing exploratory univariable and multivariable Cox regression analyses for overall survival and progression-free survival, with detailed model results provided in Supplementary Tables S3–S4. These models were performed to describe associations between selected clinical, dosimetric, and procedural variables and outcomes. Findings should be interpreted cautiously given the retrospective design, limited number of events, baseline imbalance between catheter-guidance strategies, and potential confounding by indication. The findings are hypothesis-generating. They should not be interpreted as validation of a definitive clinical dose threshold.

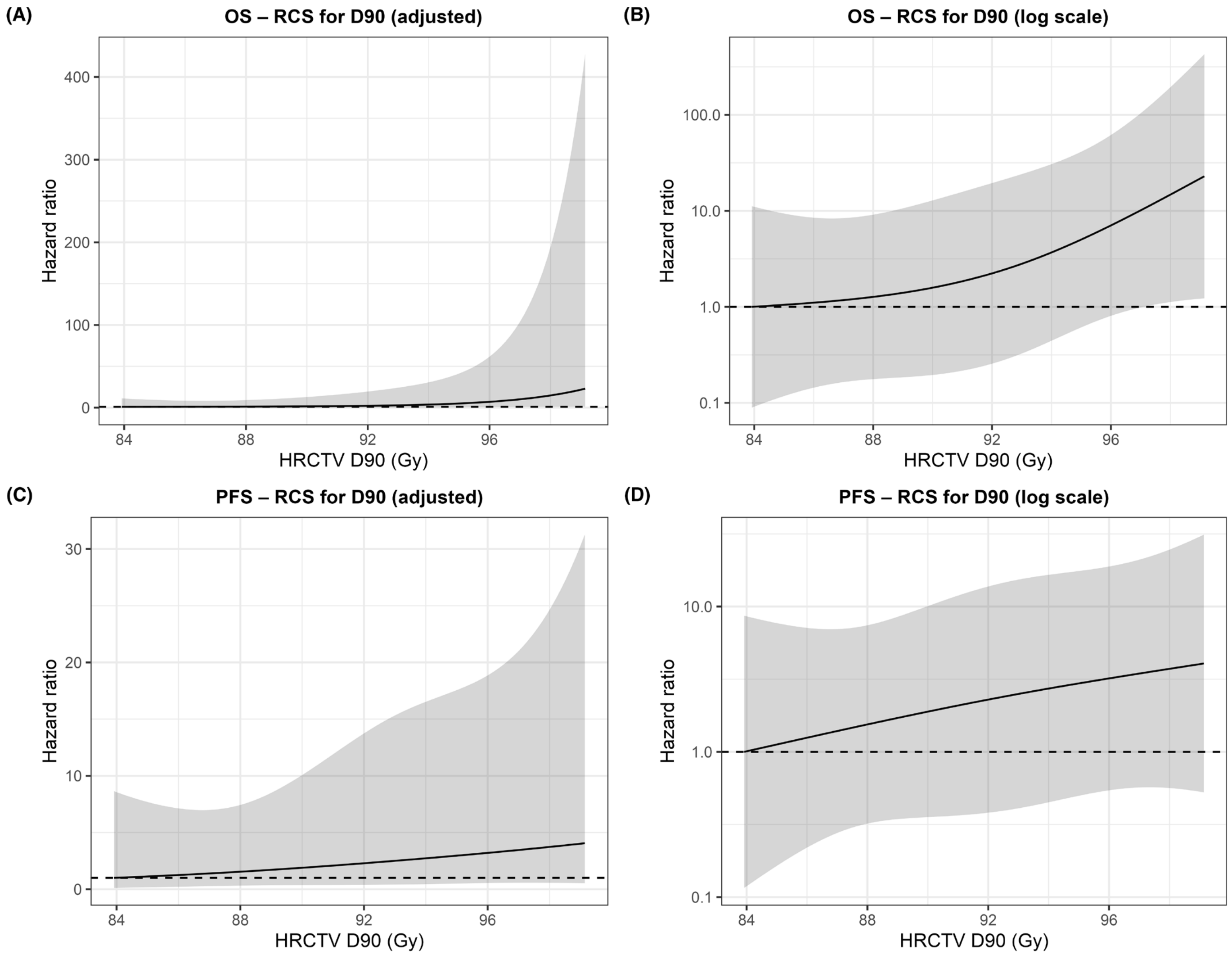

Figure S4. Exploratory restricted cubic spline analyses of the association between HR-CTV D90 and survival outcomes. Restricted cubic spline models illustrating possible non-linear associations between HR-CTV D90 and overall survival or progression-free survival. These analyses were hypothesis-generating and were not intended to define or validate a clinical dose threshold. Findings should be interpreted cautiously given the limited number of events, follow-up maturity, and potential confounding by indication.

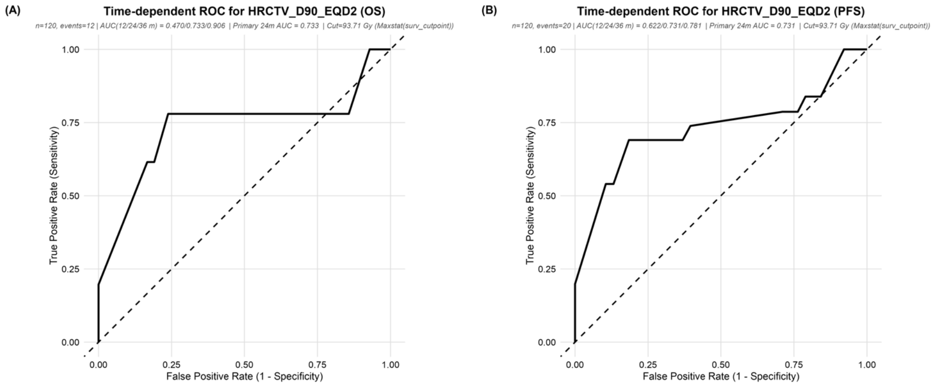

Figure S5. Time-dependent ROC curves for HR-CTV D90. Time-dependent ROC curves showing the discriminatory performance of HR-CTV D90 for overall survival and progression-free survival at 1, 2, and 3 years. These analyses are exploratory and should not be interpreted as definitive threshold-validation evidence. The findings are hypothesis-generating.

Table S1. Baseline and dosimetric characteristics according to straight-channel and curved-channel guidance strategies, with standardized mean differences (SMDs).

Variable	Straight-channel guidance (n = 60)	Curved-channel guidance (n = 60)	SMD
Age (years), median (Q1–Q3)	57.9 (49.3–62.3)	58.1 (46.2–61.9)	0.03
FIGO stage 2018, n (%) IB3–IIB / IIIA–IVA	16 (26.7%) / 44 (73.3%)	2 (3.3%) / 58 (96.7%)	0.65
Tumor size (cm), median (Q1–Q3)	5.3 (4.1–6.2)	5.9 (4.8–7.0)	0.35
Tumor size ≤6 / >6 cm, n (%)	43 (71.7%) / 17 (28.3%)	33 (55.0%) / 27 (45.0%)	0.34
Histology (SCC / non-SCC)	57 (95.0%) / 3 (5.0%)	56 (93.3%) / 4 (6.7%)	0.07
Overall treatment time (weeks)	8.2 (7.9–9.2)	8.3 (6.9–9.1)	0.04
HR-CTV volume (cm^3)	46.6 (36.9–55.8)	65.0 (55.3–77.1)	0.83
HR-CTV D90 (EQD2 Gy, α/β = 10)	89.9 (89.2–95.5)	93.8 (89.2–99.1)	0.48
V100 (%)	41.0 (31.1–50.6)	49.6 (44.0–58.8)	0.52
COIN	0.7 (0.7–0.8)	0.7 (0.6–0.8)	0.06
DHI	0.5 (0.4–0.5)	0.5 (0.4–0.5)	0.00
EBRT EQD2 (Gy, α/β = 10)	49.6 (44.2–49.6)	49.6 (44.2–49.6)	0.00
Brachytherapy EQD2 (Gy, α/β = 10)	45.9 (39.7–47.7)	49.6 (39.7–49.6)	0.27
Rectum D2cc EQD2 (total Gy, α/β = 3)	72.0 (67.2–74.7)	73.4 (69.9–75.2)	0.20
Bladder D2cc EQD2 (total Gy, α/β = 3)	74.5 (70.3–79.5)	77.9 (74.4–82.2)	0.36
Sigmoid D2cc EQD2 (total Gy, α/β = 3)	65.1 (59.3–69.2)	64.7 (61.3–70.3)	0.04
Bowel D2cc EQD2 (total Gy, α/β = 3)	62.3 (56.6–68.2)	63.6 (59.9–67.4)	0.10

Values are presented as median (Q1–Q3) or number (percentage). Straight-channel guidance and curved-channel guidance were selected according to residual target geometry, pelvic anatomy, and anticipated catheter feasibility within the same patient-specific template-guided adaptive workflow. These categories were not intended as balanced comparative treatment groups. An SMD > 0.10 was considered indicative of meaningful imbalance, and an SMD > 0.25 was considered indicative of substantial imbalance. Abbreviations: SMD = standardized mean difference; SCC = squamous cell carcinoma; EQD2 = equivalent dose in 2 Gy fractions; HR-CTV = high-risk clinical target volume; COIN = conformity index; DHI = dose homogeneity index. SMDs were used to describe baseline and dosimetric imbalance and were not used for formal hypothesis testing.

Table S2. Additional dosimetric parameters for image-guided adaptive brachytherapy.

Parameter	All patients (n = 120)	Straight-channel guidance	Curved-channel guidance
HR-CTV D90 (Gy/fx)	6.3 (5.8–6.7)	6.4 (5.9–6.8)	6.1 (5.7–6.5)
DNR	0.5 (0.5–0.6)	0.5 (0.5–0.6)	0.5 (0.5–0.6)
SIB EQD2 to nodes (Gy, α/β = 10)	60.6 (55.9–60.6)	60.6 (55.9–60.6)	60.6 (55.9–60.6)
EBRT EQD2 to OARs (Gy, α/β = 3)	48.4 (43.2–48.4)	48.4 (43.2–48.4)	48.4 (43.2–48.4)
Rectum D2cc EQD2 (BT, Gy, α/β = 3)	25.8 (22.5–27.0)	24.9 (22.2–28.1)	26.4 (23.5–26.8)
Bladder D2cc EQD2 (BT, Gy, α/β = 3)	30.1 (25.3–34.5)	27.5 (23.8–32.5)	32.0 (26.5–35.7)
Sigmoid D2cc EQD2 (BT, Gy, α/β = 3)	18.4 (13.4–22.2)	17.6 (13.3–21.5)	19.0 (13.4–23.2)
Bowel D2cc EQD2 (BT, Gy, α/β = 3)	16.9 (12.4–20.5)	16.4 (11.6–20.2)	17.2 (13.2–21.7)

Values are presented as median (Q1–Q3) for all patients and stratified by catheter-guidance strategy. Straight-channel guidance and curved-channel guidance were selected according to residual target geometry and pelvic anatomy; these data are descriptive and should not be interpreted as a causal

comparison between guidance strategies.

Abbreviations: EQD2 = equivalent dose in 2 Gy fractions; HR-CTV = high-risk clinical target volume; DNR = dose non-uniformity ratio.

Table S3. Exploratory univariable Cox regression analyses for overall survival and progression-free survival.

Endpoint	Variable	HR	95% CI	P value	Sig
OS	Age (per 10 years increase)	0.91	0.56–1.47	0.697	
OS	FIGO stage (IIIA–IVA vs IB3–IIB)	6.56	0.34–>20.00	0.076	·
OS	Tumor size (>6 cm vs ≤6 cm)	3.47	1.04–11.53	0.042	*
OS	Nodal status (N+ vs N0)	1.21	0.38–3.81	0.748	
OS	Histology (Non-SCC vs SCC)	1.18	0.15–9.16	0.876	
OS	Catheter-guidance strategy (curved-channel vs straight-channel)	2.05	0.61–6.85	0.243	
OS	HR-CTV D90 (per 5 Gy increase)	2.44	1.11–5.35	0.026	*
OS	EBRT dose EQD2 (per 5 Gy increase)	0.74	0.26–2.14	0.578	
OS	Brachy dose EQD2 (per 5 Gy increase)	0.74	0.26–2.14	0.578	
OS	COIN (per 0.1 increase)	0.96	0.68–1.36	0.821	
OS	Overall treatment time (per 0.5 week increase)	1.08	0.86–1.36	0.508	
PFS	Age (per 10 years increase)	0.95	0.65–1.39	0.792	
PFS	FIGO stage (IIIA–IVA vs IB3–IIB)	9.32	0.52–>20.00	0.021	*
PFS	Tumor size (>6 cm vs ≤6 cm)	2.24	0.93–5.41	0.073	·
PFS	Nodal status (N+ vs N0)	1.01	0.41–2.48	0.981	
PFS	Histology (Non-SCC vs SCC)	0.8	0.11–5.95	0.824	
PFS	Catheter-guidance strategy (curved-channel vs straight-channel)	1.22	0.49–3.06	0.671	
PFS	HR-CTV D90 (per 5 Gy increase)	1.51	0.94–2.44	0.087	·
PFS	EBRT dose EQD2 (per 5 Gy increase)	1.01	0.42–2.40	0.986	
PFS	Brachy dose EQD2 (per 5 Gy increase)	1.01	0.42–2.40	0.986	
PFS	COIN (per 0.1 increase)	1.01	0.88–1.16	0.857	
PFS	Overall treatment time (per 0.5 week increase)	1	0.85–1.17	0.996	

"*" indicates P < 0.05; "·" indicates borderline significance (0.05 ≤ P < 0.10).

Hazard ratios (HRs) and 95% confidence intervals (CIs) are shown to two decimal places. Values with very wide upper bounds are displayed as ">20.00" and should be interpreted with caution due to the limited number of events. Catheter-guidance strategy was selected according to case complexity and should not be interpreted as a balanced comparative treatment allocation.

Abbreviations: OS = overall survival; PFS = progression-free survival; HR = hazard ratio; CI = confidence interval; FIGO = International Federation of Gynecology and Obstetrics; SCC = squamous cell carcinoma; HR-CTV = high-risk clinical target volume; D90 = minimum dose delivered to 90% of the target volume; EBRT = external beam radiotherapy; EQD2 = equivalent dose in 2 Gy fractions; COIN = conformity index; OTT = overall treatment time; Gy = gray.

Table S4. Exploratory multivariable Cox regression analyses for overall survival and progression-free survival.

Endpoint	Variable	HR	95% CI	P value	Sig
OS	Age (per 10 years increase)	1.19	0.66–2.14	0.562	
OS	FIGO stage (IIIA–IVA vs IB3–IIB)	2.94	0.13–>20.00	0.424	
OS	Tumor size (>6 cm vs ≤6 cm)	2.02	0.52–7.92	0.273	
OS	Nodal status (N+ vs N0)	0.58	0.16–2.08	0.372	
OS	Histology (Non-SCC vs SCC)	1.83	0.25–13.16	0.549	
OS	HR-CTV D90 (per 5 Gy increase)	2.31	0.93–5.77	0.066	·

OS	Overall treatment time (per 0.5 week increase)	1.03	0.82–1.30	0.767	
PFS	Age (per 10 years increase)	1.11	0.73–1.69	0.615	
PFS	FIGO stage (IIIA–IVA vs IB3–IIB)	6.65	0.34–>20.00	0.099	·
PFS	Tumor size (>6 cm vs ≤6 cm)	1.67	0.63–4.42	0.280	
PFS	Nodal status (N+ vs N0)	0.73	0.28–1.87	0.492	
PFS	Histology (Non-SCC vs SCC)	1.41	0.24–8.38	0.708	
PFS	HR-CTV D90 (per 5 Gy increase)	1.37	0.80–2.33	0.270	
PFS	Overall treatment time (per 0.5 week increase)	0.99	0.84–1.16	0.866	

"*" indicates $P < 0.05$; "·" indicates borderline significance ($0.05 \le P < 0.10$).

All covariates were entered by force; Firth's penalized likelihood method was applied when the events-per-variable ratio (EPV) was < max(10, 2 × k). Proportional-hazards (PH) assumptions were tested only for Cox models fitted by the standard method. Findings should be interpreted as exploratory because of the retrospective design, limited number of events, and potential confounding by indication. Abbreviations: OS = overall survival; PFS = progression-free survival; HR = hazard ratio; CI = confidence interval; PH = proportional hazards; EPV = events per variable; FIGO = International Federation of Gynecology and Obstetrics; SCC = squamous cell carcinoma; HR-CTV = high-risk clinical target volume; D90 = minimum dose delivered to 90% of the target volume; EBRT = external beam radiotherapy; EQD2 = equivalent dose in 2 Gy fractions; COIN = conformity index; OTT = overall treatment time; Gy = gray.
